# Splice-Switching Antisense Oligonucleotides Reduce LRRK2 Kinase Activity in Human LRRK2 Transgenic Mice

**DOI:** 10.1016/j.omtn.2020.06.027

**Published:** 2020-06-27

**Authors:** Joanna A. Korecka, Ria Thomas, Anthony J. Hinrich, Alyssa M. Moskites, Zach K. Macbain, Penelope J. Hallett, Ole Isacson, Michelle L. Hastings

**Affiliations:** 1 Neuroregeneration Research Institute, McLean Hospital, Harvard Medical School, Belmont, MA 02478, USA; 2 Center for Genetic Diseases, Chicago Medical School, Rosalind Franklin University of Medicine and Science, North Chicago, IL 60064, USA

**Keywords:** LRRK2, antisense oligonucleotide, Parkinson’s disease, LRRK2 BAC mice, splicing, RAB10

## Abstract

Parkinson’s disease (PD) is a progressive neurological disorder estimated to affect 7–10 million people worldwide. There is no treatment available that cures or slows the progression of PD. Elevated leucine-rich repeat kinase 2 (LRRK2) activity has been associated with genetic and sporadic forms of PD and, thus, reducing LRRK2 function is a promising therapeutic strategy. We have previously reported that an antisense oligonucleotide (ASO) that blocks splicing of *LRRK2* exon 41, which encodes part of the kinase domain, reverses aberrant endoplasmic reticulum (ER) calcium levels and mitophagy defects in PD patient-derived cell lines harboring the *LRRK2* G2019S mutation. In this study, we show that treating transgenic mice expressing human wild-type or G2019S *LRRK2* with a single intracerebroventricular injection of ASO induces exon 41 skipping and results in a decrease in phosphorylation of the LRRK2 kinase substrate RAB10. Exon 41 skipping also reverses LRRK2 kinase-dependent changes in LC3B II/I ratios, a marker for the autophagic process. These results demonstrate the potential of *LRRK2* exon 41 skipping as a possible therapeutic strategy to modulate pathogenic LRRK2 kinase activity associated with PD development.

## Graphical Abstract

**Figure:**
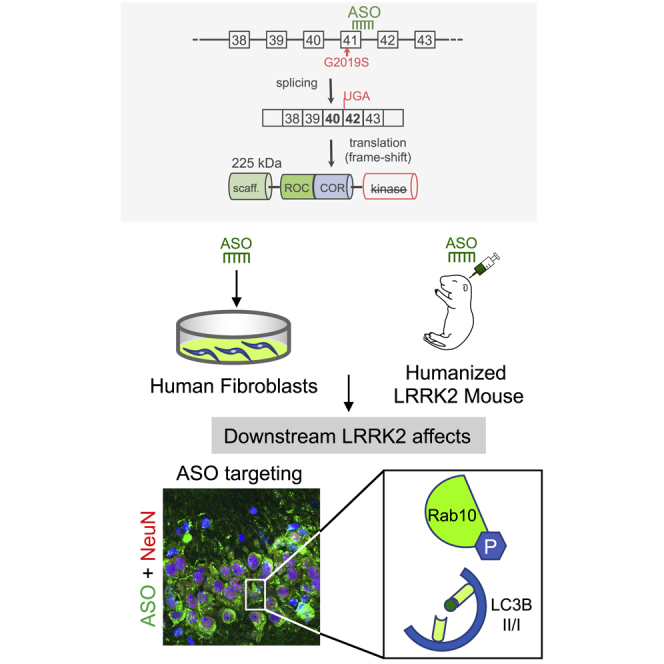

## Teaser

Splice-switching antisense oligonucleotides (ASOs) targeting a Parkinson’s disease (PD)-associated LRRK2 variant were tested in human-derived fibroblasts and transgenic mice. One ASO dose in mice resulted in long-term LRRK2 targeting and decreased RAB10 phosphorylation and LC3B II/I ratios, suggesting LRRK2 functional changes, and promoting ASOs as a therapeutic strategy for PD.

## Introduction

Parkinson’s disease (PD) is one of the most common neurodegenerative diseases. Progressive and debilitating, PD is a movement disorder characterized by tremor, muscular rigidity, and slow and uncontrollable movement. These symptoms are associated with loss of dopaminergic neurons in the substantia nigra and deficiency of the neurotransmitter dopamine. Medications that manage symptoms exist, although none halts or slows the disease progression.

The majority of PD cases are idiopathic, with ∼10% caused by known genetic mutation.^1^ The most common genetic risk factors associated with PD, accounting for 1%–2% of all PD cases, occur in the leucine-rich repeat kinase 2 (*LRRK2*) gene, encoding a serine/threonine protein kinase. These mutations are dominantly inherited and mostly cause hyperactivation of the LRRK2 kinase. A G2019S mutation within the LRRK2 kinase domain is the most common mutation associated with PD.^2^ Studies have also reported an increased risk of sporadic PD development with genetic variations in the *LRRK2* locus,^3^ as well as increased LRRK2 kinase activity levels in sporadic PD postmortem brain tissue.^4^ This evidence of a pathogenic role for LRRK2 in PD supports a therapeutic strategy targeting LRRK2 kinase activity for a large portion of PD patients. Drug discovery efforts targeting LRRK2 have been primarily focused on small-molecule LRRK2 kinase inhibitors, with phase I clinical trials ongoing for two such candidates (ClinicalTrials.gov: NCT03710707 and NCT04056689). Current inhibitors are focused on achieving greater specificity and better potency, as early-stage studies showed significant toxicity in the lungs and kidneys of treated rats and non-human primates, highlighting potential side effects of this peripheral dosing strategy with small-molecule inhibitors.^5^ Furthermore, there is evidence that some pharmacological kinase inhibitors are less efficient *in vivo* at decreasing LRRK2 kinase activity when the increase is due to the G2019S mutation.^6^ Nonetheless, if successful, such pharmacology-based kinase inhibitors will likely be of great value for PD patients. However, it is important to consider alternative strategies in order to increase the likelihood of creating an effective treatment for PD.

Antisense oligonucleotides (ASOs) have proven to be effective at specifically modulating gene expression for therapeutic benefit. In particular, an ASO drug to treat the pediatric neurodegenerative disease spinal muscular atrophy (SMA), called nusinersen (Spinraza), has an excellent safety profile when delivered directly to the central nervous system (CNS) and has shown dramatic therapeutic efficacy in patients.^7^^,^^8^ Nusinersen is an ASO that base pairs to its target RNA and alters precursor (pre-)mRNA splicing by creating a steric block that prevents binding of RNA splicing proteins. Given its clinical success for SMA, this strategy holds promise for drug development for other neurodegenerative diseases.

We have recently reported on an ASO-based approach to downregulate pathogenic LRRK2 expression in PD patient-derived cells. In these studies, we used two strategies to target LRRK2 expression. First, we designed a splice-switching ASO that induced *LRRK2* exon 2 skipping that resulted in an open-reading frameshift early in the mRNA, effectively reducing LRRK2 expression levels.^9^ Second, we designed an ASO that induced skipping of *LRRK2* exon 41, which houses the G2019S mutation within the kinase domain.^9^^,^^10^ Both of these ASO strategies resulted in normalization of mitophagy rates in PD patient-derived fibroblast cells.^9^ ASO-based skipping of *LRRK2* exon 41 also normalized altered endoplasmic reticulum (ER) calcium levels in PD patient induced pluripotent stem cell (iPSC)-derived neurons.^10^ These results provide important *in vitro* support for an ASO-based exon 41 skipping strategy capable of rescuing LRRK2-dependent cellular dysfunction, with a potential to correct PD-associated cellular toxicity.

In this study, we tested our ASO-based *LRRK2* exon 41 (ASO 41-1) skipping strategy in transgenic *LRRK2* full-length wild-type (WT) and *LRRK2* G2019S bacterial artificial chromosome (BAC) mice and show that ASO 41-1 induces exon 41 skipping in multiple areas of the brain for up to 8 weeks after a single intracerebroventricular (i.c.v.) injection. Both of the *LRRK2* BAC mice have elevated LRRK2 levels and LRRK2 kinase activity, as evidenced by an increase in phosphorylation of its substrate RAB10.^11^^,^^12^ LC3B II/I ratios, a marker for autophagosome load and/or autophagic flux, is also perturbed in the mice, likely as a consequence of LRRK2 overexpression, a phenotype also observed in human fibroblasts carrying the *LRRK2* G2019S mutation.^9^ ASO-mediated exon 41 skipping decreases phosphorylation of RAB10 and normalizes LC3B II/I ratios in the brain tissue of these mice. Taken together, our results here, in mice, and from our previous work in PD cells in *vitro* provide evidence that targeting LRRK2 exon 41 splicing with ASOs has a therapeutic effect on pathogenic LRRK2 expression and thus may be a promising drug candidate for treating PD in humans.

## Results

### ASOs Block Splicing of LRRK2 Exon 41 in PD Patient-Derived Fibroblasts

**Figure 1:**
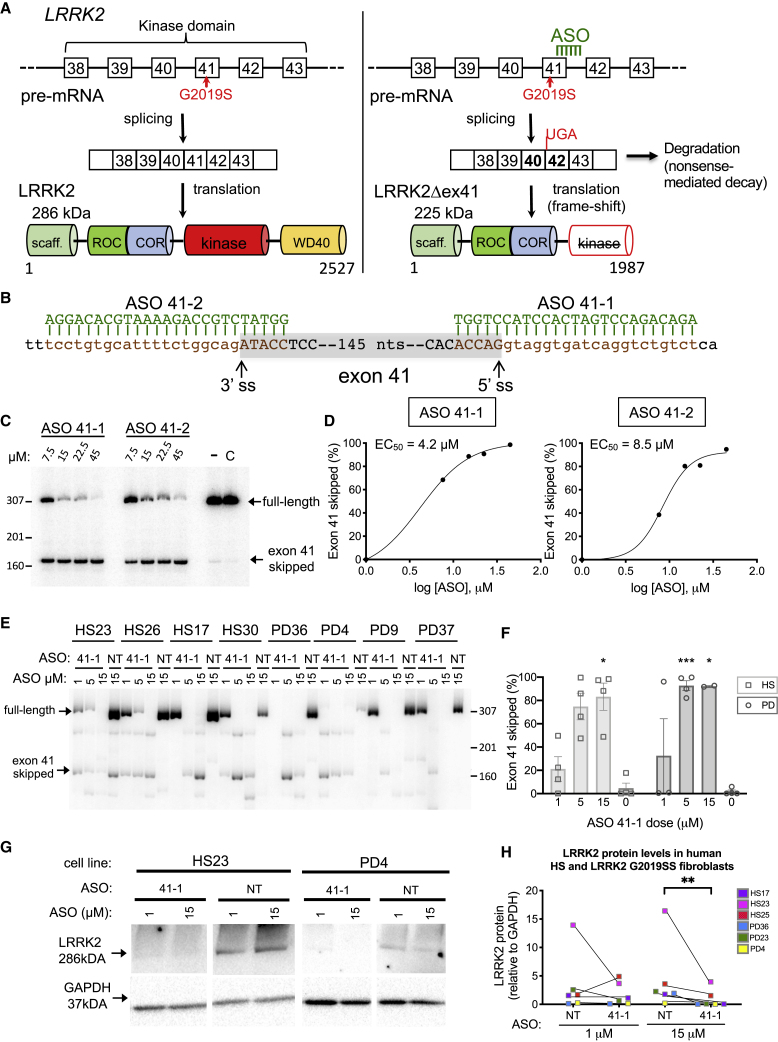
ASOs Block Splicing of *LRRK2* Exon 41 in Human Fibroblast Cell Lines (A) The *LRRK2* G2019S mutation results in the production of a dominant negative form of LRRK2 with elevated protein kinase activity. This aberrant activity is associated with Parkinson’s disease (PD) in patients with this mutation. The downregulation or inhibition of LRRK2 G2019S can be achieved by blocking splicing of exon 41, causing a frameshift in *LRRK2* mRNA, resulting in either a nonsense-mediated decay (NMD) of the unstable pre-mRNA, or formation of a truncated protein lacking kinase activity. ASO-induced *LRRK2* exon 41 skipping is predicted to mitigate disease symptoms by lessening the toxic effects of the mutated LRRK2 protein. In the pre-mRNA, boxes represent exons and lines introns. LRRK2 protein domains and the predicted molecular weight (kDa) of full-length LRRK2 and LRRK2 lacking exon 41 (LRRK2Δex41) are shown. The number of amino acids in the LRRK2 isoforms is shown beneath the protein model. (B) Sequence and targeting position of ASOs 41-1 and 41-2 and the complementary sequence in exon 41. Lowercase letters indicate intronic sequence and uppercase letters represent a partial sequence of the 161 nt of exon 41 (shaded gray). The 3′ and 5′ splice sites (ss) are indicated. (C) RT-PCR analysis of *LRRK2* RNA from a human PD patient fibroblast cell line, PD37, transfected with ASOs targeting exon 41. The spliced products are indicated. Endo-Porter-treated only (−) and untreated (C) samples are included as controls. (D) Quantitation of results from (C). The graph (right) represents the percent of exon 41 skipped [(exon 41 skipped/exon 41 skipped + full-length) × 100] in relationship to the log of the dose. The ASO potency was determined by calculating the half-maximal inhibitory concentration (EC_50_) after fitting the data using nonlinear regression with a variable slope. (E) Electrophoretic analysis of RT-PCR amplicons of RNA isolated from human fibroblast cell lines with WT (HS23, HS26, HS17, HS30) or G2019S *LRRK2* (PD36, PD4, PD9, PD37) treated with the indicated concentrations of ASO 41-1 targeting exon 41 for skipping or a non-targeted (NT) control ASO. (F) Quantitation of exon 41 skipping from (E). 0 value indicates NT control ASO. Error bars are SEM. ∗p < 0.05, ∗∗∗p < 0.001, by mixed effects analysis (restricted maximum likelihood [REML]) with Tukey’s multiple comparison test (MCT). (G) LRRK2 protein detection in cell lysates isolated either from healthy subject controls (HS23 representative line) or PD patient fibroblasts carrying the *LRRK2* G2019S mutation (PD4 representative line) treated with 1 or 15 μM of the NT control ASO or the ASO targeting exon 41 (41-1). (H) Western blots were quantified for LRRK2 protein levels normalized to GAPDH as a loading control. Western blots were performed twice in two independent experiments. Blots of all samples are shown in Figure S6. Error bars are SEM. ∗p < 0.05, by Friedman’s test with Dunn’s MCT.

Pre-mRNA splicing requires consensus sequences, called splice sites, at the junctions of the exons and introns. The 5′ splices site (ss) or donor site is located at the end of the exon (3′ end) and beginning (5′ end) of the intron, and the 3′ splice site or acceptor site is at the end of the intron (3′ end) and beginning (5′ end) of the following exon. These sites are critical for recognition of the exons by the splicing machinery, which catalyzes the cleavage of the intron and ligation of exons to form the mRNA for translation. Given the importance of the 5′ and 3′ splice sites in the recognition of an exon, blocking the sequences by hybridization of an ASO to the region is a way to mask the sites from recognition by the splicing machinery and cause skipping of the exon and thereby exclusion from the resulting mRNA. Thus, we designed two ASOs, 41-1 and 41-2, complementary to the 5′ and 3′ splice site regions of *LRRK2* exon 41, respectively (Figures 1A and 1B). The exclusion of exon 41 from *LRRK2* mRNA results in a reading frameshift and the introduction of a premature termination codon (PTC) in exon 42. The creation of a PTC in exon 42 is expected to induce nonsense-mediated mRNA decay (NMD). Any mRNA escaping NMD will encode a 225-kDa protein truncated at its C terminus by 540 amino acids (aa), relative to the 286-kDa (2,527-aa) full-length LRRK2 protein (Figure 1A).

To assess ASOs for their ability to induce exon 41 skipping *in vitro*, ASO 41-1 or 41-2 was transfected into *LRRK2* G2019S PD patient-derived fibroblast cells and after 48 h RNA was collected and analyzed for *LRRK2* exon 41 skipping by reverse transcriptase polymerase chain reaction (RT-PCR). A dose-dependent increase in exon 41 skipping was observed for both ASOs (Figure 1C). ASO 41-1 was more potent (half-maximal inhibitory concentration [EC_50_] of 4.2 μM) than ASO 41-2 (EC_50_ of 8.5 μM) (Figure 1D). ASO 41-1 induced nearly complete exon 41 skipping in multiple fibroblast cell lines derived from unaffected healthy subjects (HSs) and PD patients compared to cells treated with a control, non-targeted (NT) ASO (Figures 1E and 1F). We observed variable levels of *LRRK2* mRNA among the cell lines as well as a basal level of exon 41 skipping in control-treated cells, suggesting that a low level of exon 41 skipping may occur naturally. Notably, *LRRK2* mRNA lacking exon 41 appeared to be unstable in these cell lines. In order to determine whether the *LRRK2* mRNA with exon 41 skipped was being degraded by NMD as a result of the PTC introduced by the reading frameshift, we treated cells with puromycin prior to collection of RNA in order to block NMD. Indeed, the amount of *LRRK2* mRNA lacking exon 41 was elevated under these conditions, indicating that the mRNA is an NMD substrate (Figure S1).

ASO-mediated exon 41 skipping and the resulting decrease in *LRRK2* full-length mRNA corresponded with a greater than 50% decrease in LRRK2 protein in the cells treated with the highest dose of ASO 41-1 compared to NT ASO treatment (Friedman stat = 12.0, p = 0.0023, Dunn’s multiple comparison test [MCT] p = 0.0035, Figures 1G and 1H), confirming our previous observations where exon 41 skipping induced a decrease in LRRK2 protein in human fibroblasts and iPSC-derived neurons.^9^^,^^10^ We did not detect an ∼225-kDa truncated LRRK2 band in the ASO 41-1-treated fibroblast samples. Taken together, these results suggest that an ASO inducing exon 41 skipping lowers LRRK2 expression in primary human fibroblast cells.

### Broad Distribution and Long-Lasting Activity of ASO 41-1 in Human LRRK2 Transgenic Mice

Next, we tested ASO 41-1 *in vivo* using transgenic mice expressing human *LRRK2* WT or *LRRK2* G2019S gene in a BAC construct. To confirm LRRK2 overexpression, we first compared LRRK2 expression in the transgenic and WT mice. Analysis of *LRRK2* mRNA using pan-*LRRK2* primers confirmed that the LRRK2 transgenic mice have a greater than 4-fold elevation of *LRRK2* RNA compared to non-transgenic littermates, which corresponded to a similar increase in LRRK2 protein, as previously described (Figures S2A and S2B).^13^, ^14^, ^15^, ^16^ Additionally, immunoblot analysis of protein homogenates from the brains of the mice revealed an increase in the phosphorylation of LRRK2 S935 (pLRRK2), a marker used for LRRK2 kinase activity^11^^,^^17^^,^^18^ (Figure S2B). The specificity of the pLRRK2 antibody was confirmed by treating non-transgenic, human *LRRK2* (*hLRRK2*) WT and *hLRRK2* G2019S brain homogenates with lambda phosphatase, which eliminated pLRRK2 detection (Figure S3). RAB10, a Ras-related GTP-binding protein with a diverse role in intracellular vesicle trafficking, is a well-characterized primary substrate of LRRK2 kinase.^11^^,^^12^ In both *in vitro* *LRRK2* WT overexpression models and *in vivo* *LRRK2* mutation knockin models, phosphorylation of RAB10 at the Th73 site is reported to be LRRK2 kinase-dependent.^11^^,^^12^^,^^19^^,^^20^ Brain homogenates from both mouse strains showed elevated phosphorylation of RAB10 at Th73 (Figure S2B), supporting a functional downstream effect of human LRRK2 overexpression in these mice.

We next tested whether ASO 41-1 could induce *hLRRK2* exon 41 skipping in the *hLRRK2* BAC mice. *hLRRK2* WT BAC neonates were injected with ASO 41-1 or a control, NT ASO by i.c.v. injection at postnatal day 2 (P2) and RNA and protein were isolated from tissue collected at P21. Spliced products were confirmed by sequence analysis of cDNA from the striatum (Figure S4A and S4B). RT-PCR analysis of RNA isolated from the striatum and hippocampus revealed that an average of 17% and 26% of *LRRK2* mRNA lacked exon 41, respectively, 3 weeks after treatment (Figure S4C). This level of exon 41 skipping corresponded with a significant decrease of LRRK2 protein in the hippocampus of the *hLRRK2* WT mice (Figure S4D, p = 0.008, t = 3.9, degrees of freedom [df] = 6).

**Figure 2:**
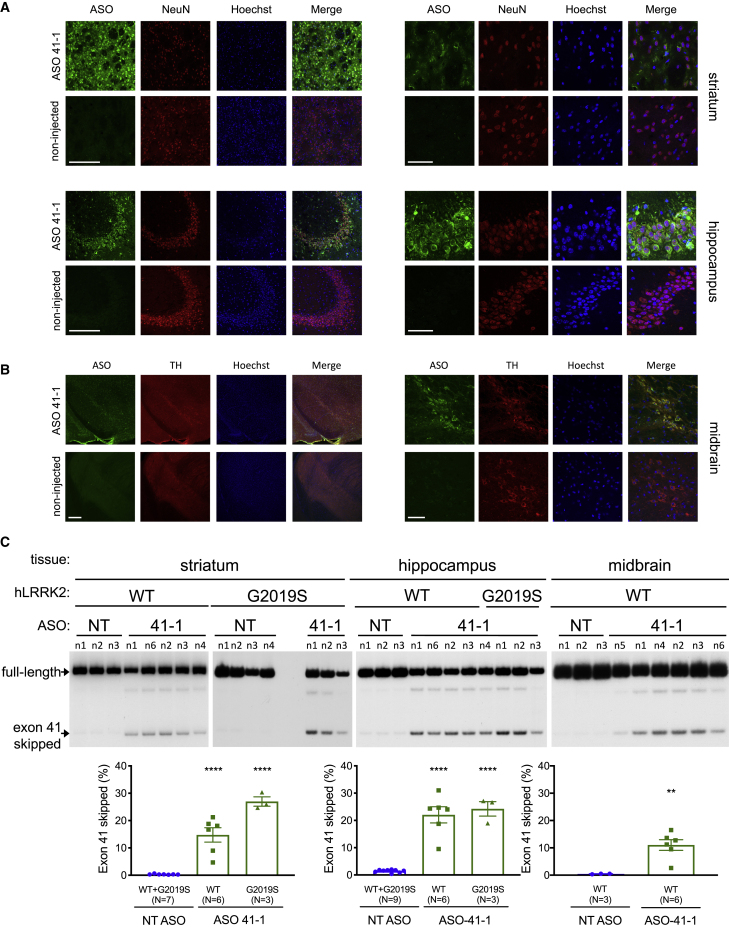
ASO 41-1 Has Long-Lasting Activity in Brain Tissue of Human *LRRK2* Transgenic Mice (A) Immunohistochemical analysis of striatum and hippocampus from P56 LRRK2 G2019S BAC mice treated with an ASO 41-1 or non-injected at P2. Coronal sections were labeled with antibodies specific to the ASO (green) and neurons (NeuN, red), stained for nuclei (Hoechst, blue), and imaged at ×20 (left) and ×63 (right). Scale bars represent 200 μm (×20) and 50 μm (×63). (B) Immunohistochemical analysis of midbrain from the same mice as in (A). Sections were labeled with antibodies specific to ASO (green) and tyrosine hydroxylase (TH, red) and imaged at ×10 (left) and ×60 (right). Scale bars represent 200 μm (×10) and 50 μm (×60). (C) RT-PCR analysis of striatum (left), hippocampus (center), and midbrain (right) from 8-week-old WT or G2019S BAC (G2019S) mice treated at P2 with 40 μg of 2′MOE ASO targeting *LRRK2* exon 41 (ASO 41-1) or non-targeted (NT ASO). Error bars are SEM. ∗∗p < 0.01, by unpaired, two-tailed t test; ∗∗∗∗p < 0.0001, one-way ANOVA with Dunnett’s MCT.

The long half-life of ASOs in the CNS allows for extended targeting with a single dose.^21^^,^^22^ We reasoned that a prolonged decrease in LRRK2 kinase activity in transgenic mice would have the most robust effects on LRRK2 function. Thus, we tested whether ASO 41-1 could induce *hLRRK2* exon 41 skipping 8 weeks after a single i.c.v. injection of P2 mice. Previous reports using an ASO-specific antibody have demonstrated that ASOs delivered in this manner distribute throughout the CNS and in a wide range of cell types.^23^, ^24^, ^25^, ^26^ Similarly, immunohistochemical analysis of ASO distribution in different brain regions of ASO-treated *hLRRK2* BAC mice revealed the presence of ASO 41-1 and NT ASO (Figures 2A, 2B, and S5A) in the hippocampus, striatum, and midbrain. ASOs co-localized with the neuronal protein NeuN (RBFOX3) (Figures 2A and S4) and dopamine neuron marker tyrosine hydroxylase (TH) (Figure 2B), demonstrating that ASOs localized to neurons in these regions. The distribution of ASO 41-1 in the brain 2 months after injection corresponded with ∼10%–25% *LRRK2* exon 41 skipping in the striatum, hippocampus, and the midbrain, indicating a long-lasting effect of the ASOs in the CNS (Figure 2C).

### ASO 41-1 Treatment Decreases LRRK2-Dependent RAB10 Phosphorylation

Having achieved ASO-induced *hLRRK2* exon 41 skipping in the mouse brain for up to 2 months after a single i.c.v. dosing, we next assessed whether these molecular changes correlate with a decrease in LRRK2 protein and phosphorylation of RAB10, a kinase substrate.^11^

**Figure 3:**
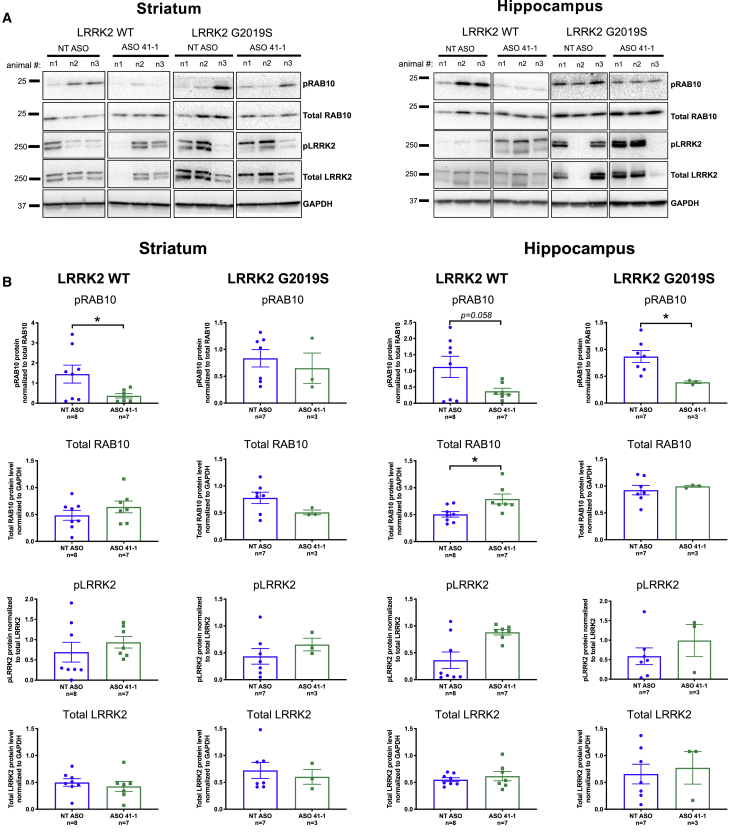
pRAB10 and LRRK2 Expression Analysis in Mouse Striatal and Hippocampal Tissue after *LRRK2* Exon 41 ASO Treatment (A) Representative western blot images of T73 phosphorylated RAB10 (pRAB10), total RAB10, S935 phosphorylated LRRK2 (pLRRK2), total LRRK2, and GAPDH detected in the striatum and hippocampus of 2-month-old *hLRRK2* WT and *hLRRK2* G2019S BAC overexpressing mice injected with either the non-target ASO (NT ASO) or the LRRK2 exon 41 ASO (ASO 41-1) at P2. For images of all samples, see Figure S6. (B) Quantification analysis of the blots represented in (A) and in Figure S6. Western blots were performed twice in two independent experiments. The specificity of the phospho-RAB10 antibody was previously described in Lis et al.,^20^ whereas the specificity of the phospho-LRRK2 antibody is shown in Figure S3. Error bars are SEM. ∗p < 0.05, ∗∗p < 0.01, by either a Student’s t test or Mann-Whitney test, depending on the normality of the data distribution.

ASO 41-1 treatment resulted in a significant reduction of RAB10 Th73 phosphorylation in the striatum of the *hLRRK2* WT mice (p = 0.047, t = 2.2, df = 13) and the hippocampus of the *hLRRK2* G2019S mice (p = 0.027, t = 2.69, df = 8), as well as a trend of decreased RAB10 phosphorylation in the hippocampus of the *hLRRK2* WT mice (p = 0.058, t = 2.08, df = 13) (Figures 3A and 3B). Total RAB10 abundance was not affected by the ASO treatment with the exception of hippocampal LRRK2 WT levels (p = 0.015, t = 2.8, df = 13). Neither pLRRK2 levels nor full-length LRRK2 protein abundance was altered by *LRRK2* ASO 41-1 treatment compared to the NT ASO-injected animals (Figures 3A and 3B). The LRRK2 antibody (UDD3) recognizes the first 883 aa of LRRK2 protein and thus should detect both the full-length and truncated isoforms, although it is unclear whether the two isoforms can be resolved and distinguished from each other. Taken together with the mRNA skipping analysis (Figure 3), these results demonstrate effective ASO 41-1 LRRK2 target engagement and functional downstream effects on RAB10 phosphorylation.

### ASO 41-1 Treatment Corrects LRRK2-Dependent Abnormalities in Autophagic Flux *In Vivo*

**Figure 4:**
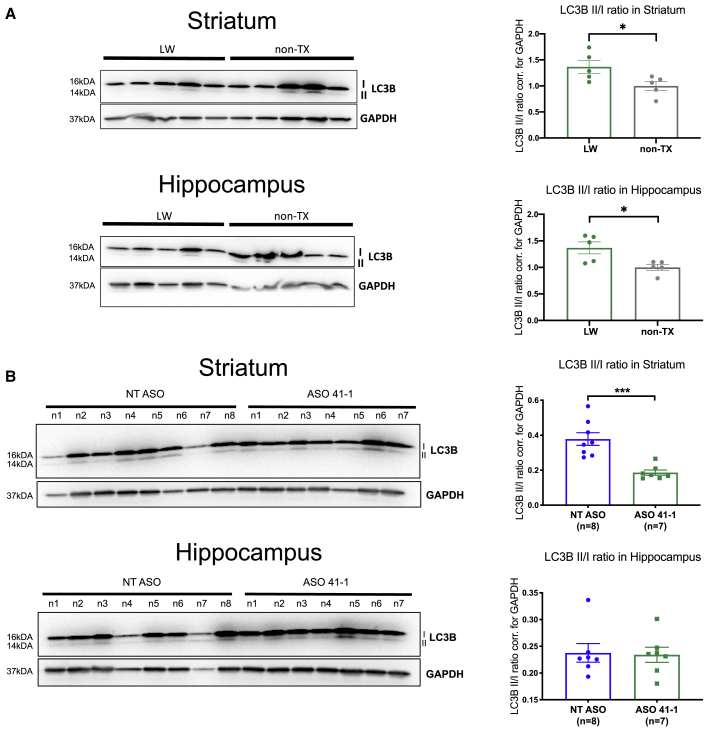
LC3B II/I Ratio Analysis in Non-transgenic and *hLRRK2* WT BAC Mice (A) LC3B protein expression analysis in 2-month-old *hLRRK2* WT BAC (LW) and wild-type non-transgenic (non-TX) mouse striatal and hippocampal tissue. Representative images of LC3B and GAPDH western blots. Quantification of LC3B II/I ratios in non-TX and LW mouse striatum and hippocampus from three independent blots. (B) Western blot images of LC3B II and I, and GAPDH proteins and quantification of LC3B II/I ratio in the striatum and hippocampus of 2-month-old *hLRRK2* WT BAC overexpressing mice injected with either the non-targeted ASO (NT ASO) or the LRRK2 exon 41 ASO (ASO 41-1). Pool of two independent blots. Error bars are SEM. ∗p < 0.05, ∗∗p < 0.01, ∗∗∗ < 0.001, by either a Student’s t test or Mann-Whitney test, depending on the normality of the data distribution.

Autophagy has been linked to LRRK2 function.^27^ Our previous work showed a direct effect of LRRK2 kinase activity on autophagosome maturation, as determined by LC3 II/I turnover in PD patient fibroblasts.^9^ Interestingly, upon comparing WT *hLRRK2* overexpressing mice, a model for increased LRRK2 kinase activity,^28^ to non-transgenic (non-TX) littermates, we also observe differences in the LC3B II/I ratio (Figure 4A). In both striatal (p = 0.039, t = 2.45, df = 8) and hippocampal (p = 0.019, t = 2.92, df = 8) tissues, the LC3B II/I ratio was elevated in the WT *hLRRK2* overexpressing mice. The *in vivo* LC3B II/I readout is a static reflection of one captured moment of the autophagic flux. Without a feasible possibility to pharmacologically inhibit the fusion of autophagosomes to the lysosomes in the brain, the observed changes are suggestive of LRRK2-dependent alterations either in the autophagic flux and/or in the autophagosome load, without the possibility of the distinction between the two (Figure 4A). We also observed a trend toward a decrease in LC3A II/I ratios in the WT *hLRRK2* mice compared to their non-transgenic littermates, although the difference was not significant (Figure S7A).

The observed changes in autophagic flux and/or autophagosome load in the CNS with *hLRRK2* WT overexpression *in vivo* (Figure 4A) led us to investigate whether ASO 41-1 treatment could rescue this phenotype. Indeed, in addition to the long-term functional effect of *LRRK2* exon 41 skipping on the phosphorylation levels of RAB10 (Figure 3), LC3B II/I ratios were also lower in the *hLRRK2* WT striatum tissue 2 months after ASO 41-1 treatment (Figure 4B, p = 0.0005, t = 4.65, df = 13, analysis of two independent blots). ASO 41-1 treatment also correlated with a decrease in the ratio of LC3A II/I in *hLRRK2* WT mouse striatum and hippocampus (Figure S7B). Importantly, together with the decrease in total LRRK2 levels, a significant decrease in the LC3B II/I ratios was also observed in the hippocampus of P21 mice, 3 weeks after ASO 41-1 treatment (Figure S4E, p = 0.015, t = 3.35, df = 6). These protein changes further validate the specificity of the ASO 41-1 treatment and the importance of LRRK2 function in the cellular autophagic processes. Overall, our results demonstrate effective ASO 41-1 target engagement, with the capacity to correct pathological features associated with *hLRRK2* overexpression.

## Discussion

In this study, we demonstrate that excluding exon 41 from *LRRK2* mRNA, using splice-switching ASOs that block exon 41 splicing, reduces RAB10 Th73 phosphorylation and normalizes the LC3B II/I ratios *in vivo*, potentially as a result of reduced LRRK2 kinase activity. A single neonatal LRRK2 ASO treatment has long-lasting functional effects in young adult mice, decreasing the elevated RAB10 phosphorylation associated with overexpression of LRRK2 and correcting autophagic flux and/or autophagosome load. These results support long-term target engagement capacity and efficacy of this ASO strategy in a genetic mouse model of PD.

We find that a single i.c.v. injection of mice expressing human *LRRK2* with ASO 41-1 induces stable exon 41 skipping of *LRRK2* pre-mRNA for up to 2 months post-treatment. Consequently, this intervention strategy results in a significant decrease of LRRK2 protein abundance 3 weeks post-treatment, and in RAB10 phosphorylation at the Th73 site 2 months post-treatment. Furthermore, exon 41 skipping in *hLRRK2* WT overexpressing mice, a model for increased LRRK2 kinase activity,^28^ decreased the LC3B II/I ratios, an autophagic flux and/or autophagosome load marker, evidently increased by overexpression of the *hLRRK2* gene. We have previously shown that LC3B ratios are altered in human fibroblasts carrying the kinase activity inducing G2019S mutation and could be rescued with a LRRK2 kinase inhibitor,^9^ which, along with our current *in vivo* data, strongly supports the involvement of LRRK2 kinase activity in the regulation of autophagosome load and/or autophagic flux. Indeed, recent work by Rocha et al.^29^ suggests that the macroautophagy pathway depends on LRRK2 kinase function, and *in vivo* application of LRRK2 kinase inhibitor in a rotenone PD model rescues the accumulation of the p62/SQSTM1 adaptor protein responsible for LC3 conversion. Supporting our previous *in vitro* data,^9^ current observed changes *in vivo* using the ASO-based strategy further validate the specificity of this therapeutic approach.

In our previous work we showed that LRRK2 protein abundance decreased in human fibroblast lines and iPSC-derived neurons following treatment with ASO 41-1, resulting in a rescue of mitophagy rate^9^ and ER calcium levels,^10^ respectively. In the current study, we observed a decrease in LRRK2 protein in the mouse brain 3 weeks after i.c.v. injection of ASO 41-1, further confirming ASO 41-1 target engagement *in vivo*. We hypothesized that the open-reading frameshift and protein truncation resulting from the exclusion of exon 41 *LRRK2* mRNA would result in the production of a kinase-dead LRRK2 protein truncated at its C terminus, if the mRNA is not degraded by NMD. In support of this hypothesis, a similar kinase-dead truncated LRRK2 protein, generated by frameshift mutations introduced into iPSCs by CRISPR-Cas9-directed gene editing, was recently shown to alter kinase-dependent cellular phenotypes.^30^ Despite achieving up to 30% exon 41 skipping in some tissues treated with ASO 41-1 and nearly 100% skipping in human fibroblast cell lines, we did not observe a band corresponding to a truncated LRRK2 on our SDS gels using a polyclonal antibody recognizing both proteins. It is possible that the antibody is not sensitive enough to detect the truncated protein in the tissues, which is less abundant than the full-length protein. NMD of the *LRRK2* mRNA lacking exon 41, which is apparent in the fibroblast cells, may also contribute to the lack of truncated protein detection. In addition, the truncated form of LRRK2 protein may be unstable due to altered structure and/or altered protein interactions,^31^^,^^32^ or due to the introduction of other destabilizing features such as a degron, shown before to occur in other alternatively spliced protein isoforms.^33^

At 2 months after ASO injection, in the more metabolically stable environment of the adult brain, we identified modifications in the direct LRRK2 kinase substrate, RAB10, but did not detect a decrease in total LRRK2 protein abundance in *hLRRK2* transgenic mice. A degree of variability in total LRRK2 protein levels in different brain regions of the 2-month-old ASO-treated mice was observed, which may contribute to the lack of consistent decrease in LRRK2 protein abundance following ASO 41-1 treatment. Changes in LRRK2 protein levels during development^34^ and/or protein stability in the adult brain, or variance in LRRK2 expression between brain regions and cell types^35^ may all contribute to this variability. It is also possible that the truncated kinase-dead form of LRRK2 is present in the adult mouse brain but cannot be resolved from the full-length protein by immunoblotting, leading to the lack of an observed change in total LRRK2 protein levels, as both are high-molecular-weight species, especially if the truncated form undergoes an extensive phosphorylation. Reliably specific antibodies to the LRRK2 c-terminus (2,069–2,087 aa), which would allow us to detect only the full-length LRRK2, are not available.^36^ Another possible explanation for the observed downstream effects on RAB10 phosphorylation and LC3B II/I ratios, despite not seeing total LRRK2 protein changes in the 2-month-old brain, may lay in alterations of the LRRK2 protein-protein interactions with GTPases and 14-3-3 proteins, regulating LRRK2 kinase activity.^4^^,^^14^^,^^31^ An ASO 41-1-generated LRRK2 truncated protein may disrupt the homeostasis of these known interactions, decreasing kinase activity. Finally, by generating a kinase-dead LRRK2 variant, we may be reducing p62 phosphorylation necessary for initiation of LRRK2 degradation via autophagy, therefore altering carefully balanced p62-LRRK2 autophagy signaling homeostasis.^37^

In the current study, we demonstrate that ASO 41-1, capable of rescuing LRRK2 cellular dysfunction *in vitro*,^9^^,^^10^ also successfully targets LRRK2 in young and adult mouse brain after single-dose treatment. Future studies will focus on better understanding the dynamics of LRRK2 expression and ASO distribution in the adult and aged CNS, as well as optimization of ASO treatment, including multiple dosing paradigm applications, which are currently tested in the clinic to achieve stable high-level target engagement and therapeutic efficacy.^38^^,^^39^

An ASO-based strategy to stably and locally decrease LRRK2 kinase activity offers an attractive alternative to pharmacological kinase inhibitors. ASO technology has evolved rapidly to overcome many of the hurdles that limited their utility as drugs in the past. To date, there have been six ASO drugs commercially approved for the treatment of humans. Among these are two splice-switching ASOs, one of which, nusinersen (Spinraza), is delivered intrathecally to treat the lethal neurodegenerative disorder SMA.^7^^,^^8^ The success of splice-switching ASOs in treating SMA supports their use for the treatment of other neurological disorders.

Ribonuclease H (RNase H)-directed ASOs (gapmers) are another type of ASO that has been commercially successful. Gapmers have a DNA core that, when base-paired with its RNA target, becomes a substrate of endogenous RNase H, which cleaves RNA that is duplexed with DNA. Through this cleavage activity, RNase H-directed ASOs degrade their target RNA and effectively downregulate gene expression. This type of ASO has recently been investigated for its potential in downregulating LRRK2 in PD. Zhao et al.^40^ showed that RNase H ASOs targeting *LRRK2* can reduce LRRK2 mRNA and protein and decrease pathological S129 phosphorylated alpha-synuclein levels in the midbrain of non-transgenic mice that had been injected with alpha-synuclein protein fibrils into the striatum. This treatment causes wire-hanging motor deficits and a reduction in TH^+^ neurons in the substantia nigra in mice, both of which were prevented by pre-treatment with an i.c.v. injection of a LRRK2 gapmer ASO. Although this study was performed in a non-transgenic mouse model that does not have pathological LRRK2 kinase activity, it demonstrates the promise that reduction of LRRK2 may have in rescuing pathology similar to that seen in PD. However, unlike the splice-switching ASO tested in the present study, inducing partial skipping and inhibition of kinase domain encoding exon 41 (containing the G2019S mutation in familial PD patients), the gapmer strategy causes a significant strong reduction in total LRRK2, which may have a negative impact on the normal activity of LRRK2 protein in the cell. With LRRK2 linked to mitochondrial health, autophagy, intracellular transport, calcium homeostasis, endocytosis, the trans-Golgi network, and microtubular structure,^27^ a complete silencing of its function may be undesirable. Also important for therapeutic development considerations, unlike splice switching ASOs, no gapmers have been US Food and Drug Administration (FDA) approved for the treatment of a neurological disorder via direct delivery to the CNS.

A single i.c.v. injection of ASO 41-1 in murine neonates results in significant skipping of exon 41 in *LRRK2* mRNA, a decrease in LRRK2 protein levels 3 weeks after treatment, and a decrease in downstream targets of LRRK2 kinase activity at 2 months post-treatment, as observed by both the decreased phosphorylation of RAB10 and altered LC3B II/I ratio. Whether these long-term functional changes are due to decreased LRRK2 protein levels or the generation of a kinase-dead truncated form of LRRK2 remain to be determined. Exon 41 skipping can alter LRRK2 function either through destabilization of the mRNA by NMD of the PTC-containing *LRRK2* mRNA lacking exon 41, and/or through the generation of the kinase-dead isoform. A similar such kinase-dead isoform was recently shown to alter LRRK2 kinase-dependent cellular phenotypes.^30^ In addition, LRRK2 has been reported to function as a dimer;^41^^,^^42^ thus, if the exon 41-skipped *LRRK2* mRNA is translated into a truncated protein, its interaction with an equal amount of full-length LRRK2 could create a non-functional dimer, resulting in a more dramatic cumulative reducing effect on LRRK2 kinase activity through this dominant negative activity. Normalizing the pathologically elevated LRRK2 kinase activity to more physiological levels while minimally affecting LRRK2 protein levels may contribute to conservation of other non-kinase-related LRRK2 protein functions such as nuclear envelope integrity.^43^ As we further progress toward a better understanding of the LRRK2 mechanism in cellular function, it is important to carefully consider the strategy and the magnitude of LRRK2 kinase manipulation. Future studies will focus on ASO dose optimization for *LRRK2* exon 41 skipping efficiency for clinical application.

Herein, we outline a clinically relevant, locally administered intervention strategy for PD based on LRRK2 kinase inhibition. Intrathecal injection is becoming more commonly used in the clinic for cancer and pain amendment, and ASO intrathecal delivery has been successful for SMA^7^^,^^8^ and Batten’s disease,^38^ with new treatments currently being clinically tested for neurological diseases such as Huntington’s disease,^44^ amyotrophic lateral sclerosis (ALS), and Alzheimer’s disease (ClinicalTrials.gov) among others. With the striking success of ASO-based therapeutic strategy development, current research is focusing on new ASO delivery methods into the CNS, including oral, subcutaneous, and even intranasal delivery routes.^45^ From this perspective, local administration into the CNS of highly specific and stable exon 41 skipping ASOs with a potential for therapeutic effect on pathogenic LRRK2 kinase activity is an attractive treatment strategy for PD patients. It is our hope that with an increase in the development of diverse treatment interventions, the dialog between a patient and the physician upon diagnosis can soon turn away from standard symptomatic treatment protocols to multiple, potential disease-halting strategies, a dramatic change for patients and their prognosis.

## Materials and Methods

### Fibroblast Cell Lines, Cell Culture, and ASO Transfection

HS and PD patient-derived fibroblast cell lines with the *LRRK2* G2019S mutation were obtained from the Coriell and the NIA Aging Cell biorepositories (Table 1). Some of the HSs and the PD cell lines were previously described and studied in detail for LRRK2-dependent mitochondrial and mitophagy dysfunction.^9^^,^^46^ Human fibroblasts were grown in HyClone DMEM/high-glucose media supplemented with 10% fetal bovine serum (FBS). Fibroblasts were transfected with ASO 41-1 (5′-AGACAGACCTGATCACCTACCTGGT-3′) or ASO 41-2 (5′-GGTATCTGCCAGAAAATGCACAGGA-3′ modified with phosphorodiamidate morpholinos (PMOs) (Gene Tools, Philomath, OR, USA) using Endo-Porter according to the manufacturer’s protocol (Gene Tools). An NT ASO PMO with no targets in the human genome was used as a negative control (5′-CCTCTTACCTCAGTTACAATTTATA-3′, Gene Tools). Cells were grown for either 48 or 72 h after transfection, and then RNA and protein were collected. Basic local alignment search tool (BLAST) analysis of ASO targets in the human genome reveals no sequences within functional RNA sequences with more than 70% complementarity to the ASO sequence.

**Table 1:** Detailed Clinical Information of the Healthy Subject and PD Patient-Derived Fibroblasts Used in the Current Study

Cell ID	Coriell ID	Description	Sex	Age at Autopsy
HS17	AG06010	healthy subject	F	62
HS23	AG11743	healthy subject	F	76
HS25	AG06241	healthy subject	M	61
HS26	AG04061	healthy subject	M	66
HS30	AG04355	healthy subject	M	67
PD4	SC1007	*LRRK2* G2019S	not documented	not documented
PD9	ND35367	*LRRK2* G2019S homo.	M	60
PD23	SC1012	*LRRK2* G2019S	not documented	not documented
PD36	–	*LRRK2* G2019S homo.	M	55
PD37	–	*LRRK2* G2019S homo.	M	74

Fibroblast lines were previously described in Korecka et al.^9^ and Smith et al.^46^ HS, healthy subject; PD, Parkinson’s disease; M, male; F, female; homo., homozygous.

### Animals

All experimental procedures involving animals were performed in accordance with Institutional Animal Care and Use Committee (IACUC) guidelines and approved by the McLean Hospital, Harvard Medical School IACUC. Animals were housed according to standard conditions, in a 12-h dark/12-h light cycle, with *ad libitum* access to food and water. Human *LRRK2* WT (B6;SJL-Tg(LRRK2)66Mjff/J) and *LRRK2* G2019S (C57BL/6J-Tg(LRRK2∗G2019S)2AMjff/J) BAC overexpressing heterogeneous mice^14^, ^15^, ^16^ were obtained from The Jackson Laboratory (Bar Harbor, ME, USA). Transgenic hemizygous mice were bred with B6SJLF1/J (for the *hLRRK2* WT colony) or C57LB/6J (for the h*LRRK2* G2019S colony) WT mice to keep the heterogeneity of the colonies. Genotypes were verified using PCR amplification of tail DNA, according to The Jackson Laboratory genotyping protocols. Genotyping was performed by the Center for Computational and Integrative Biology (CCIB) DNA Core Facility at the Massachusetts General Hospital (Cambridge, MA, USA). Both male and female mice were used in all experiments.

### Neonatal i.c.v. Injection of ASO and Mouse Tissue Collection

For neonatal i.c.v. injections, pups (P1–P2) were treated with a single 40-μg dose of 2′-*O*-methoxyethyl sugars (2′MOE) and a phosphorothioate (PS) backbone-modified NT ASO (5′-CCTCTTACCTCAGTTACA-3′) or ASO 41-1 (5′-CCTGATCACCTACCTGGT-3′) (Integrated DNA Technologies) via i.c.v. injection according to a published procedure.^22^ In brief, ASOs were diluted in sterile 0.9% saline with 0.01% Fast Green FCF, and 2.5 μL was injected into the left ventricle using a 33G needle (point style 4; angle, 30) affixed to a glass Hamilton syringe approximately 2.5 mm anterior to the lambda suture and 1 mm lateral to the sagittal suture to a depth of 2 mm. For the P21 time point, pups were treated with 25 μg of PMO NT ASO or 20 μg of 2′MOE ASO 41-1.

Transgenic hemizygote *LRRK2* WT and *LRRK2* G2019S BAC overexpressing animals were perfused with cold phosphate-buffered saline (PBS) either 3 weeks or 2 months after ASO i.c.v. injection. The brain was isolated and the left hemisphere was postfixed in 4% paraformaldehyde overnight and moved to 30% sucrose. The right hemisphere was immediately dissected and hippocampus, striatum, midbrain, and cortex tissue were all isolated, snap-frozen, and stored for analysis of exon skipping and protein expression.

### RNA Isolation and Analysis

RNA was isolated from tissue and cells in culture using TRIzol reagent (Life Technologies, Carlsbad, CA, USA) according to the manufacturer’s protocol. Tissues were homogenized in TRIzol using a power homogenizer. RNA was reverse transcribed using a GoScript reverse transcription system (Promega, Madison, WI, USA). Radiolabeled PCR was carried out as previously described^9^^,^^10^ using human-specific *LRRK2* primers (hLRRK2ex40F, 5′-CCTACAGCACAGGATTGC-3′ and hLRRK2ex42R, 5′-CCTCTACTATTCTACCTCC-3′). Primers that recognize both mouse and human LRRK2 mRNA (pan-LRRK2) were panLRRK2F, 5′-GTATCCCAATGCTGCCATC-3′ and panLRRK2R, 5′-CAACCATAGGCCATGGGGC-3′.

### Immunoblotting and Protein Expression Quantification

Mouse brain tissues were lysed in radioimmunoprecipitation assay (RIPA) buffer (Thermo Scientific, #89900) supplemented with Halt protease and phosphatase inhibitor cocktail (Thermo Fisher Scientific, #1861284) and EDTA (Thermo Fisher Scientific, #1861283) and sonicated before centrifugation. Protein concentration of the supernatant was measured with a bicinchoninic acid (BCA) assay (Pierce). 20–30 μg of protein was loaded into Criterion precast 4%–20% or 5%–8% gradient polyacrylamide gels (Bio-Rad) and size-separated using a Bio-Rad electrophoresis system. Proteins were transferred to a polyvinylidene fluoride (PVDF) membrane using a Trans-Blot Turbo system (Bio-Rad) run at 21 V and 2.5 A for 7 min, or at 25 V and 1.3 A for 15 min for LRRK2 detection, prior to blocking for 1 h with 5% protein blocker (Bio-Rad) or 5% BSA (Sigma, #A9418) in Tris-buffered saline with 0.1% Tween 20 (TBST). Membranes were then incubated overnight at 4°C with the following primary antibodies: anti-pSer935 LRRK2 (Abcam, ab133450), anti-LC3B (Cell Signaling Technology, #3868, 1:1,000), anti-phosphorylated (phospho)-RAB10 (Abcam, ab230261, 1:250), and anti-GAPDH (EMD Millipore, #AB2302, 1:5,000). After washing three times for 10 min with TBST, the membranes were incubated with horseradish peroxidase (HRP)-coupled secondary antibody for 1 h at room temperature. Following three 10-min washes with TBST, the membranes were developed using Advansta WesternBright Sirius chemiluminescent substrate (Advansta, K-12043-D20) and imaged using ChemiDoc XRS with Image Lab software (Bio-Rad). Optical density analysis for assessment of protein expression was performed with Image Lab software (Bio-Rad) and normalized to GAPDH.

For the detection of the full-length RAB10, LRRK2, and LC3A proteins, blots were stripped using stripping buffer (100 mM β-mercaptoethanol, 2% SDS, 62.5 mM Tris-HCl) for 30 min at 55°C, washed, and blocked in 5% protein blocker as described above. The RAB10 (Cell Signaling Technology, #8127, 1:500), LRRK2 (MRC, University of Dundee, monoclonal rabbit 30-12 [100–500] UDD3, 1:250), and LC3A (Cell Signaling Technology, #4599, 1:1,000) antibodies were then added to the blocking solution and incubated for 2 days at 4°C. Secondary antibody incubation and imaging were then performed as described above. For the final quantification of the phosphorylated RAB10 and LRRK2 protein levels, the intensity of the phosphorylated protein bands was normalized to the intensity of the full-length RAB10 and LRRK2 protein bands.

Dephosphorylation of LRRK2 protein was performed using lambda phosphatase enzyme (NEB, P0753S). 40 μg of cell lysate was mixed with 1 mM MnCl_2_, 1× NEB PMP buffer, and 800 U of lambda phosphatase enzyme and incubated at 30°C for 30 min. Immunoblotting was performed using the dephosphorylated samples as described above.

### Immunohistochemistry

Brains were sectioned in the coronal plane at a thickness of 40 μm using a microtome and stored in anti-freezing media. For immunohistochemistry, sections were washed in 3× PBS and blocked in 4% goat serum in 0.9% Triton X-100/PBS for 2 h at room temperature. Sections were washed in 1× PBS and incubated in primary antibody mixture (anti-ASO, 1:1,000, Ionis Pharmaceuticals; anti-NeuN, 1:1,000, Millipore, MAB 377; anti-TH, 1:500, Pel-Freez Biologicals, P60101-150) overnight at 4°C. Sections were incubated in secondary antibody mixture (goat anti-rabbit Alexa Fluor 488, 1:400, goat anti-mouse Alexa Fluor 594, 1:400, or goat anti-sheep Alexa Fluor 594, 1:400, Jackson ImmunoResearch Laboratories) for 2 h at room temperature. Sections were stained with Hoechst 33342 (1:10,000 in PBS) for 10 min and mounted on glass slides with ProLong Gold mounting media. ×20 and ×63 high-magnification confocal images were obtained with a Zeiss LSM 510 confocal microscope.

### Quantification and Statistics

Statistical data analysis was performed with GraphPad Prism software v7.0c. All data are expressed as mean ± SEM. Data were analyzed for normal distribution using the Shapiro-Wilk’s test. An unpaired two-tailed Student’s t test or ratio paired t test was used to compare two means as appropriate, or one-way ANOVA when comparing more than two means. When data were not normally distributed, statistical analysis was performed using the Mann-Whitney test or Friedman’s test. For each experiment, the specific statistical analysis used is noted in the figure legends. A p value <0.05 was considered significant for all analyses.

## Author Contributions

Conceptualization: J.A.K., R.T., M.L.H., P.J.H., and O.I.; Methodology: J.A.K., R.T., A.J.H., and M.L.H.; Investigation: J.A.K., R.T., A.J.H., A.M.M., and Z.K.M.; Writing: J.A.K., R.T., A.J.H., and M.L.H.; Visualization: J.A.K., R.T., A.J.H., and M.L.H.; Funding Acquisition: J.A.K., M.L.H., P.J.H., and O.I.; Supervision: J.A.K., M.L.H., P.J.H., and O.I.

## Conflicts of Interest

The authors declare no competing interests

## Supplemental Information

Document S1. Figures S1–S7

Document S2. Article plus Supplemental Information
