# Supplementary material for: Splice-Switching Antisense Oligonucleotides Reduce LRRK2 Kinase Activity in Human LRRK2 Transgenic Mice
Source: Mol Ther Nucleic Acids. 2020 Jun 27;21:623–35. doi: 10.1016/j.omtn.2020.06.027 (PMC7393423; doi:10.1016/j.omtn.2020.06.027)
Supplement: Document S1. Figures S1–S7 [file mmc1.pdf]

OMTN, Volume *21*

Supplemental Information

Splice-Switching Antisense Oligonucleotides Reduce LRRK2 Kinase Activity in Human LRRK2 Transgenic Mice

Joanna A. Korecka, Ria Thomas, Anthony J. Hinrich, Alyssa M. Moskites, Zach K. Macbain, Penelope J. Hallett, Ole Isacson, and Michelle L. Hastings

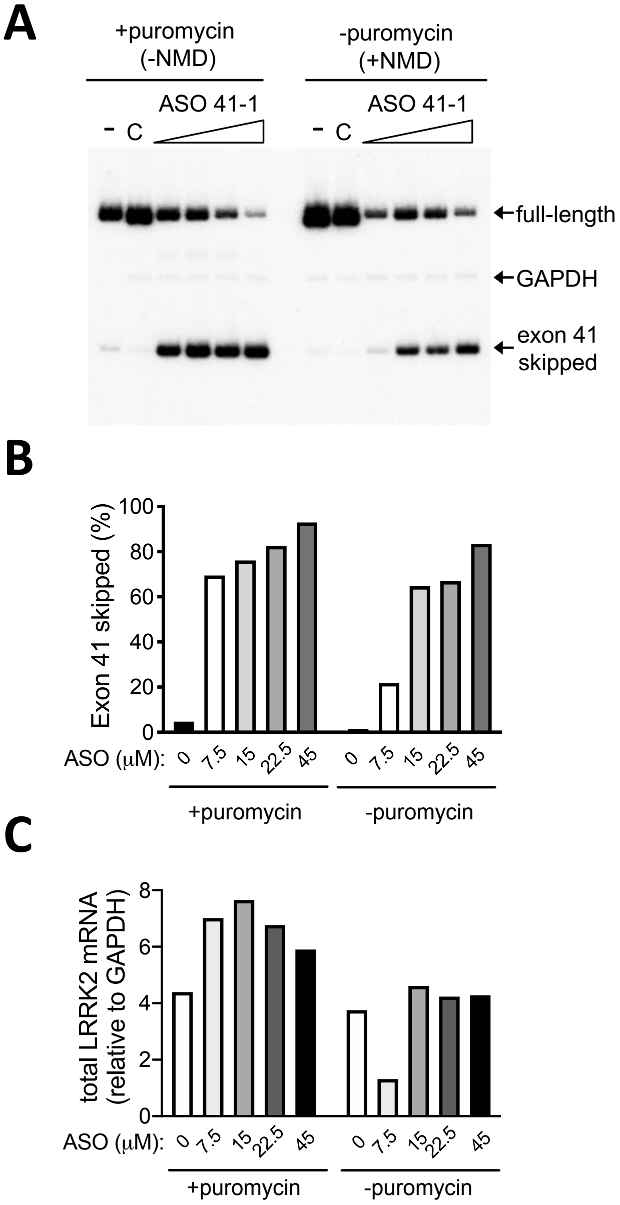

Figure S1: *LRRK2* mRNA lacking exon 41 is a substrate for nonsense mediated decay (NMD) as a result of the premature termination codon introduced by ASO-induced exon 41 skipping (See **Figure 1**). **A.** RT-PCR analysis of a patient fibroblast (PD37) cell line carrying the G2019S mutation treated with an increasing concentration of ASO (PMO) in the presence or absence of puromycin (See **Figure 1**). **B.** Quantification of the percent of *LRRK2* mRNA that has exon 41 skipped [exon 41 skipped/(exon 41 skipped+full-length *LRRK2*)]x100 from gel image in A. **C**. Quantitation of total *LRRK2* mRNA normalized to GAPDH as determined from gel image in A.

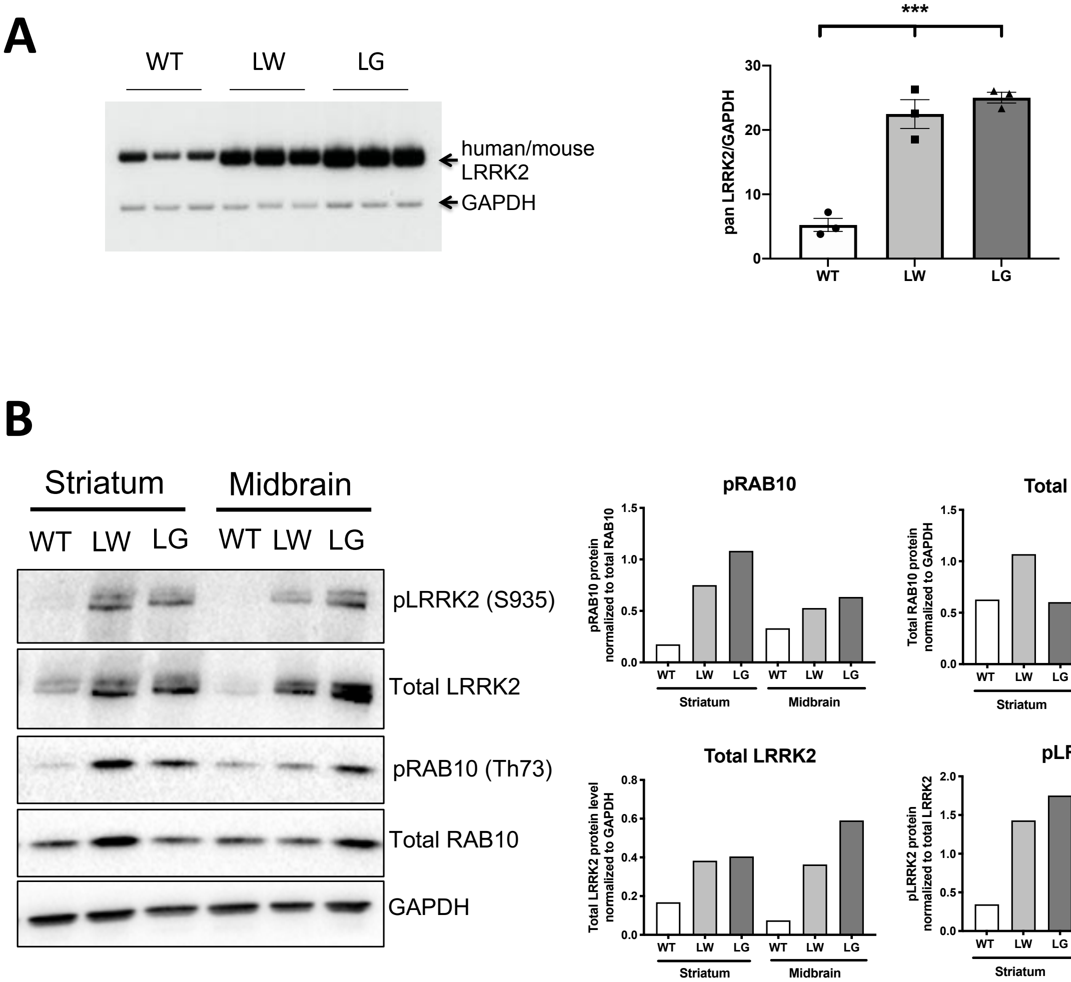

Figure S2. LRRK2 and phosphoRAB10 levels in humanized *LRRK2* WT and G2019S BAC transgenic mice (See **Figure 2** and **3)**. **A.** *LRRK2* mRNA expression in P21 hippocampal tissue from WT non-transgenic (WT), *LRRK2* WT BAC (LW), and *LRRK2* G2019S BAC (LG). **B.** Immunoblot image and quantification of pLRRK2 (S935), total LRRK2, pRAB10 (Th73) and total RAB10 protein levels in the striatum and midbrain of 2 months old WT, LW and LG mice. See **Figure 2** and **3.**

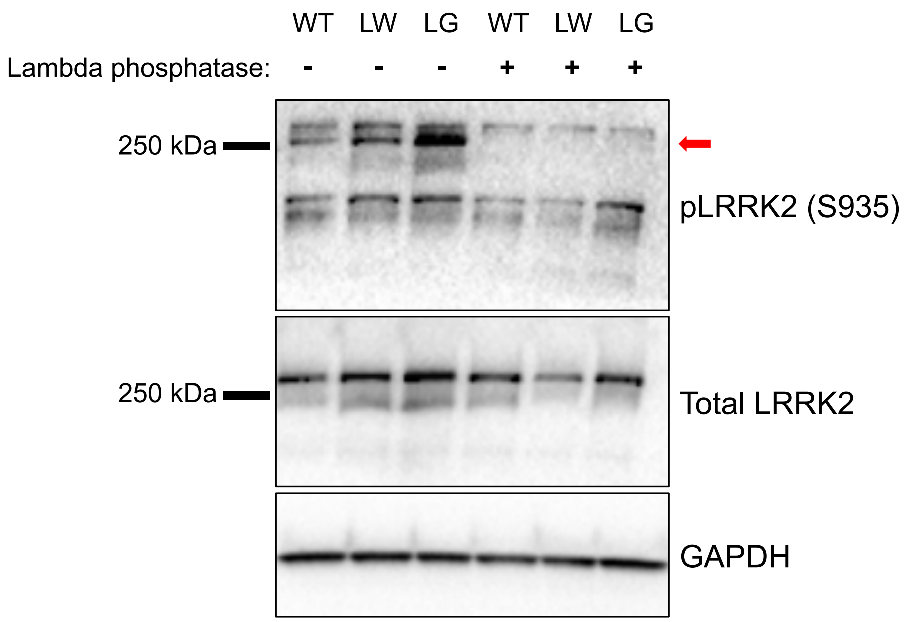

Figure S3. Specificity of pLRRK2 antibody in mouse brain tissue lysates (See **Figure S2** and **Figure 3**). Immunoblot analysis of mouse brain tissue lysates from 2 month old wild type (WT), *LRRK2* WT BAC (LW) and *LRRK2* G2019S BAC (LG) overexpressing mice treated with and without lambda phosphatase, probed for pLRRK2 (S935) and total LRRK2 proteins. Red arrow indicates the band specific for phosphorylated LRRK2 (S935). See **Figure S2** and **Figure 3**.

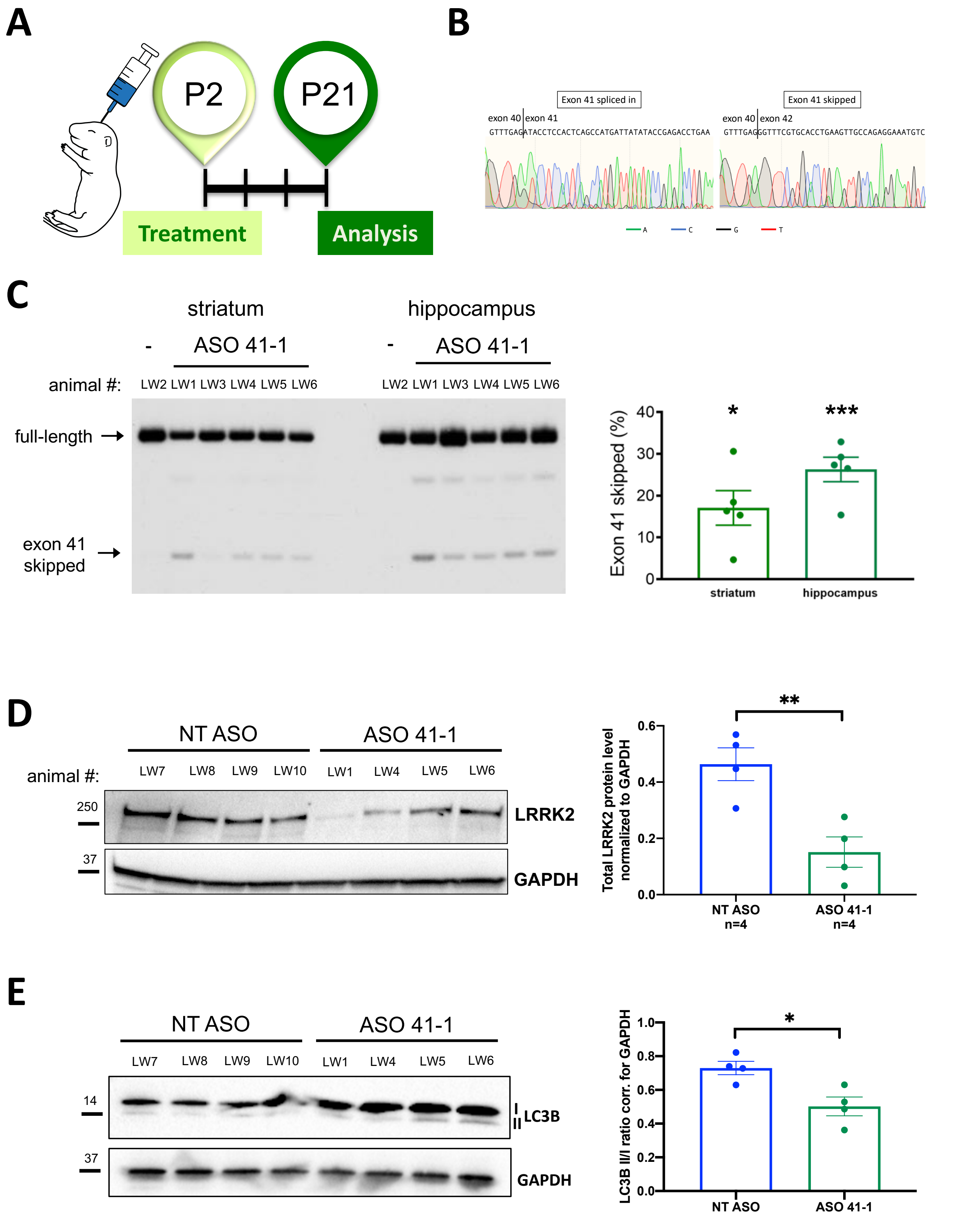

Figure S4. ASO 41-1 activity in the brain of human *LRRK2* transgenic mice at P21. **A.** Analysis of ASO 41-1 activity in brain tissues collected from P21 LRRK2 WT (LW) mice ASO treated at P2 by i.c.v. injection. **B.** Sequencing trace files from analysis of full-length and exon 41 skipped *LRRK2* amplicons. The region surrounding the exon 40/41 (full-length) and 40/42 splice junction shows the expected splicing events. **C.** RT-PCR analysis of RNA isolated from striatum and hippocampus of five different mice (animal #LW1-LW6) treated with ASO 41-1 (2'MOE, 20 µg) at P2

injection. Graph shows quantitation of PCR products in gel (left). Exon 41 skipped = [exon 41 skipped/(skipped+full-length)*100]. Error bars are SEM, *$p<0.05$; ***$p<0.001$. Statistical analysis was performed using student T test. **D**. Western blot analysis of protein lysates from hippocampus of four *LRRK2* WT BAC mice treated with ASO 41-1 (2'MOE, 20 µg) or ASO NT (PMO, 25 µg) at P2, three weeks post ASO injection. 5-8% SDS gels were stained for LRRK2 and GAPDH protein, see **Figure S6B**. **E**. LC3B analysis of protein lysates from hippocampus of four *LRRK2* WT BAC mice treated with ASO 41-1 or ASO NT at P21, three weeks post ASO injection. Error bars are SEM, *$p<0.05$; **$p<0.01$. Statistical analysis was performed using student T test.

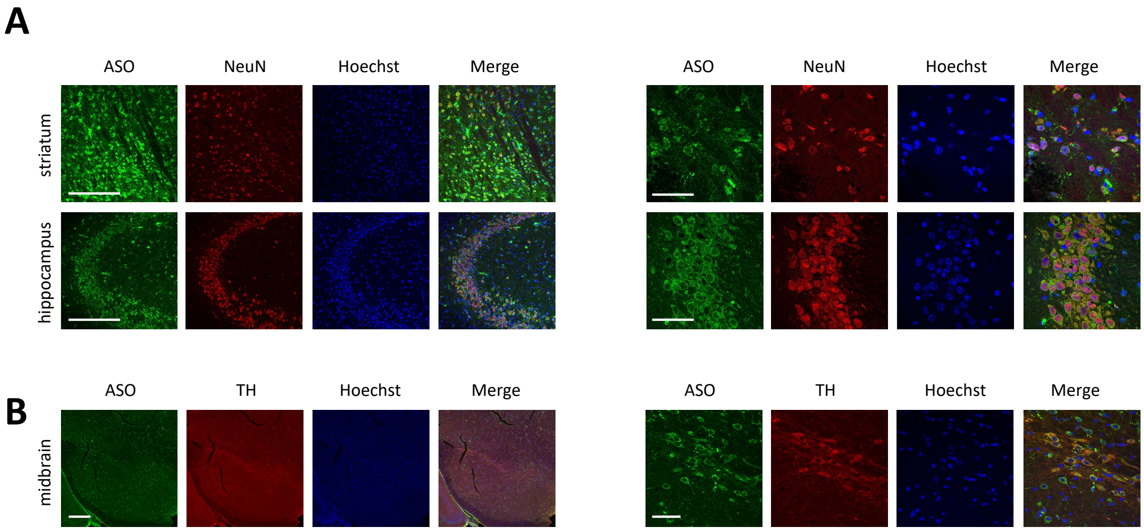

Figure S5. A. Immunohistochemical analysis of striatum and hippocampus from P56 *LRRK2* G2019S BAC mice treated with a non-targeted (NT) ASO at P2 (See **Figure 2)**. Coronal sections were labeled with antibodies specific to the ASO (green) and neurons (NeuN, red), stained for nuclei (Hoechst, blue), and imaged at 20x (left) and 63x (right). Scale bars represent 200µm (20x) and 50µm (63x). **B.** Analysis of midbrain from same mouse as A. Sections were labeled with antibodies specific to ASO (green) and tyrosine hydroxylase (TH, red), and imaged at 10x (left) and 60x (right). Scale bars represent 200µm (10x) and 50µm (60x). See **Figure 2.**

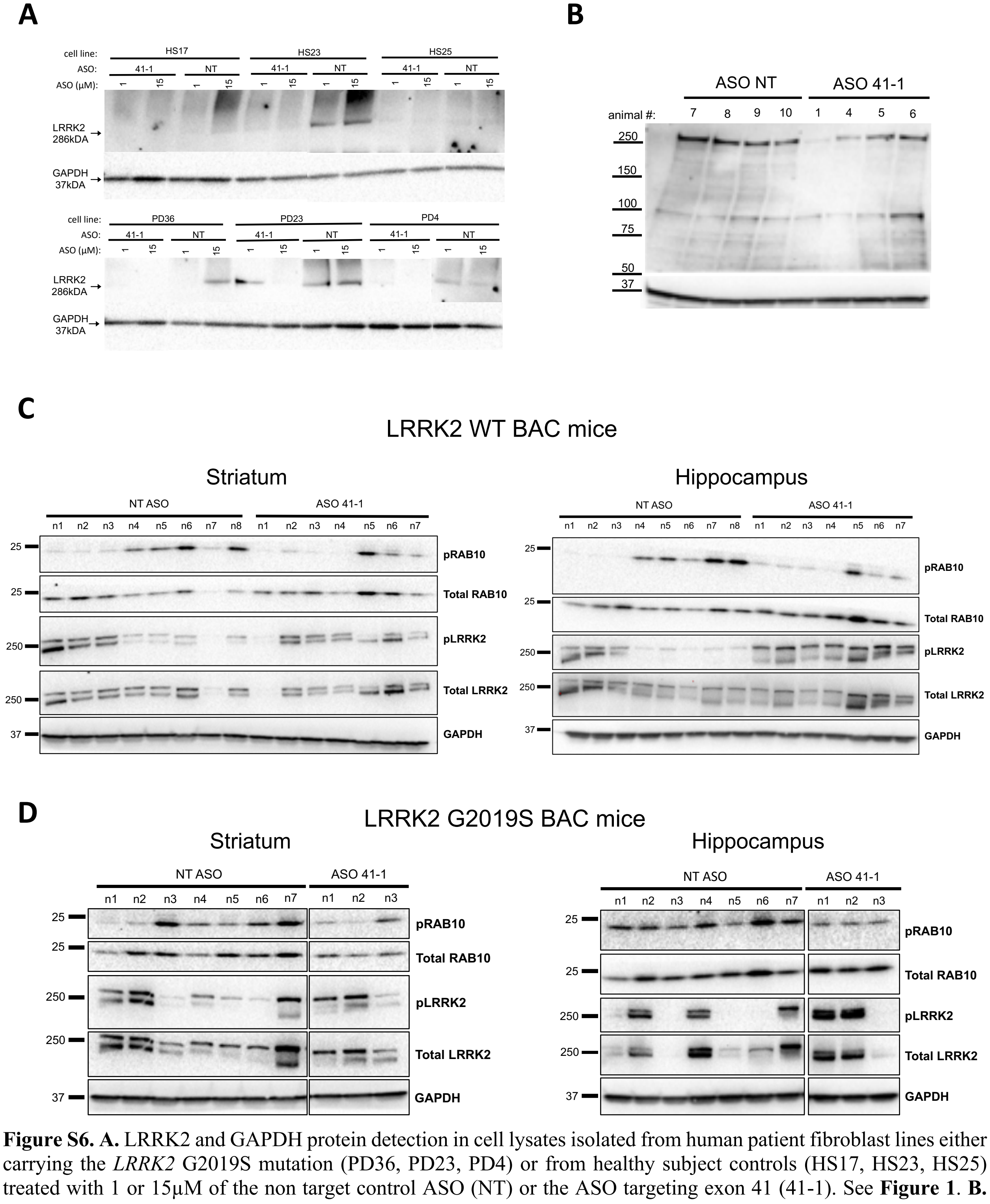

Figure S6. A. LRRK2 and GAPDH protein detection in cell lysates isolated from human patient fibroblast lines either carrying the *LRRK2* G2019S mutation (PD36, PD23, PD4) or from healthy subject controls (HS17, HS23, HS25) treated with 1 or 15µM of the non target control ASO (NT) or the ASO targeting exon 41 (41-1). See **Figure 1**. **B.** Western blot analysis of protein lysates from hippocampus of four different *LRRK2* WT mice treated with ASO 41-1 (2'MOE) or ASO NT (PMO) at P21 post neonatal P2 ASO i.c.v. injection. 5-8% SDS gels were stained for LRRK2 and GAPDH protein. See **Figure S4**. **C** and **D**. Western blot images of T73 phosphorylated Rab10 (pRab10), total Rab10, S935 phosphorylated LRRK2 (pLRRK2), total LRRK2 and GAPDH detected in the striatum and hippocampus of *LRRK2* WT BAC (**C**) or *LRRK2* G2019S BAC (**D**) overexpressing mice. Mice were i.c.v. injected at P2 with either

the non-target ASO (NT ASO) or the LRRK2 exon 41 ASO (ASO 41-1) and tissue was analyzed 2 months later at P56. See **Figure 3**.

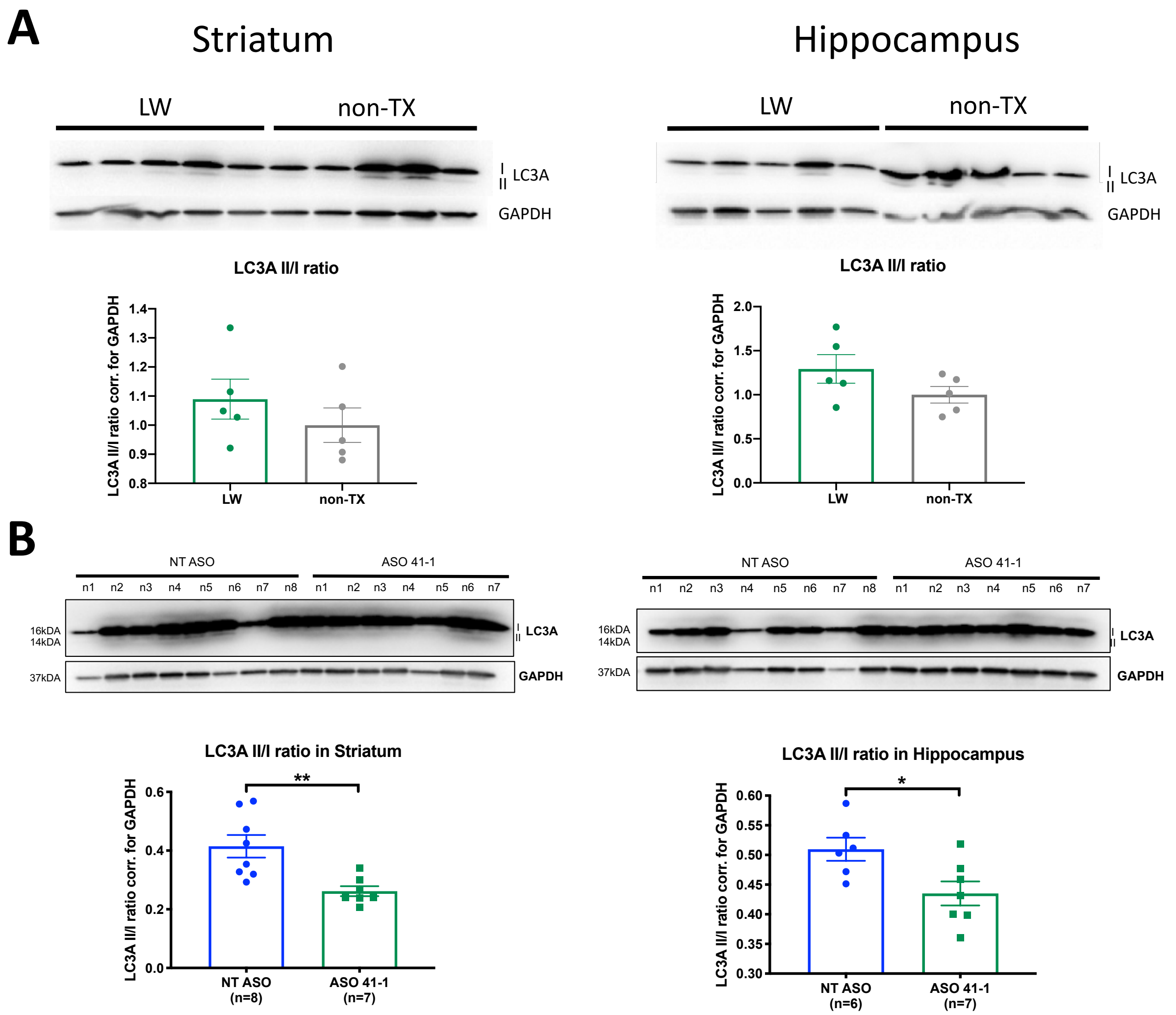

Figure S7. LC3A II/I protein ratios in non-transgenic, *hLRRK2* WT BAC overexpressing mice and in *hLRRK2* WT mice after ASO 41-1 treatment (See **Figure 4**). **A.** LC3A expression analysis in 2 month old *hLRRK2* WT BAC (LW) and non-transgenic (non-TX) mouse striatal and hippocampal tissue. Representative images of LC3A and GAPDH western blots images and quantification of LC3A II/I ratios (three independent blots) in non transgenic (non-TX) and *hLRRK2* WT BAC (LW) overexpressing mouse brains. Error bars are SEM, *p<0.05; Statistical analysis was performed using student T test. See **Figure 4**. **B.** Western blot images and quantification analysis of LC3A and GAPDH proteins detected in the striatum (pool of three independent blots) and hippocampus (pool of two independent blots) of *hLRRK2* WT BAC overexpressing mice injected with either the non-target ASO (NT ASO) or the LRRK2 exon 41 ASO (ASO 41-1) (2 months post i.c.v. injection). Error bars are SEM, *p<0.05, **p<0.01. Statistical analysis was performed using either student T test of Mann Whitney test, depending on the normality of data distribution. See **Figure 4**.
